# Copper(II) Complexes of Amino Acids and Peptides Containing Chelating bis(imidazolyl) Residues

**DOI:** 10.1155/S1565363303000104

**Published:** 2003

**Authors:** lmre Sóvágó, Katalin Ősz, Katalin Várnagy

**Affiliations:** Department of Inorganic and Analytical Chemistry University of Debrecen Debrecen H-4010 Hungary

## Abstract

Copper(II) complexes of amino acids and peptides containing the chelating bis(imidazolyl) residues have been reviewed. The results reveal that bis(imidazolyl) analogues of these biomolecules are very effective ligands for metal binding. The nitrogen donor atoms of the chelating agent are the major metal binding sites under acidic conditions. In the presence of terminal amino group the multidentate character of the ligands results in the formation of various polynuclear complexes including the ligand and the imidazole bridged dimeric species. The most intriguing feature of the coordination chemistry of these ligands is that the deprotonation of the coordinated imidazole-N(1)H groups results in the appearance of a new chelating site in the molecules. It leads to the formation of stable trinuclear complexes via negatively charged imidazolato bridges.


### Copper(II) Complexes of Amino Acids and Peptides Containing Chelating bis(imidazolyl) Residues

Imre Sóvágó*, Katalin Ősz and Katalin Várnagy

*Department of Inorganic and Analytical Chemistry, University of Debrecen, H-4010 Debrecen, Hungary*
*e-mail: sovago@delfin.klte.hu*

(Received January 31, 2003; Accepted March 6, 2003)

### ABSTRACT

Copper(II) complexes of amino acids and peptides containing the chelating bis(imidazolyl) residues have been reviewed. The results reveal that bis(imidazolyl) analogues of these biomolecules are very effective ligands for metal binding. The nitrogen donor atoms of the chelating agent are the major metal binding sites under acidic conditions. In the presence of terminal amino group the multidentate character of the ligands results in the formation of various polynuclear complexes including the ligand and the imidazole bridged dimeric species. The most intriguing feature of the coordination chemistry of these ligands is that the deprotonation of the coordinated imidazole-N(1)H groups results in the appearance of a new chelating site in the molecules. It leads to the formation of stable trinuclear complexes *via* negatively charged imidazolato bridges.

**Keywords:** amino acids, peptides, bis(imidazol-2-yl)methane, copper(II), stability constants

### INTRODUCTION

Imidazole nitrogen donor atoms are among the most common metal binding sites in metalloenzymes. The metal ion coordination generally takes place *via* the N(1) or N(3) atoms of imidazole residues as it is represented by a great variety of iron, zinc and copper proteins including myoglobin and other heme proteins, carbonic anhydrase, carboxypeptidase and blue copper proteins. Another group of metalloenzymes, however, contains the imidazole moiety as a bridging ligand; e.g. in CuZn-superoxide dismutase (CuZnSOD), where both nitrogen atoms of the negatively charged imidazolato residue take part in metal binding.

The monodentate coordination of imidazole side chains is generally modelled by the metal complexes of peptides containing histidyl residues. A great number of studies have been performed in this field and the most important observations have already been reviewed by several authors /1-5/. The results of these studies reveal that the presence of histidine in peptides significantly enhances the metal binding ability of the ligands, but the extent of the increase in stability and the structure of the various complexes largely depend on the location of histidyl residues in the peptide chain.

The metal complexes of synthetic ligands containing two or more imidazole residues are also frequently used to mimic the structure and catalytic activity of the active sites of metalloproteins /6-8/. These molecules can provide a high structural variety for metal ion coordination including both the monodentate and bridging imidazolyl coordinations. The ligand bis(imidazolyl)methane (BIM) is one of the simplest representatives of polyimidazole ligands, but it is a very strong chelating agent and its amino (BIMA) and carboxylate (BIP) derivatives can be easily attached to amino acids or peptides *via* amide bonds. The copper(II) complexes of these amino acid and peptide analogues containing the bis(imidazol-2-yl)methyl residues have been studied in our laboratories in the last few years /9-15/. In this survey we would like to give a brief account of the most important results of these studies. The data clearly represent that these synthetic derivatives of amino acids and peptides are very effective ligands for metal binding and their complex formation processes can be finely tuned by the location of the monodentate and chelating side chains of the parent biomolecules.

### EXPERIMENTAL

The synthetic procedures for the preparation of ligands have already been reported elsewhere /9-16/. The purity of the derivatives of amino acids and peptides was checked by TLC and HPLC and their structures were proved by $^1$H NMR measurements.

The protonation constants of the ligands and the stability constants of the copper(II) complexes (log $\beta_{pqr}$ for $[Cu_pH_qL_r]$) were determined by potentiometric titrations in aqueous solution, under standard conditions. Experimental details of the pH-metric measurements and calculation of the equilibrium parameters have been reported previously /14,15/. The structures of the various species formed in solution and the metal binding sites of the ligands were elucidated by UV-VIS, EPR, NMR and CD spectroscopic and MALDI-MS studies. The applications of these experimental techniques are discussed in the original publications /9-15/.

### RESULTS AND DISCUSSION

### Selection of the ligands

The parent ligand bis(imidazol-2-yl)methane (BIM) is a well known chelating agent forming stable, 6-membered chelate rings with a series of transition elements /17-20]. The amino (BIMA) and carboxylate (BIP) derivatives of BIM (see Scheme 1) made it possible to link the chelating agent to the C- or N-termini of peptides *via* the formation of an additional amide bond. The resulting ligands contain the characteristic metal binding sites of both the peptides and the chelating agent BIM. Taking into account the fact that the coordination ability of amino acids and peptides is significantly influenced by the presence of the terminal amino group and the coordinating side chain residues the synthetic analogues of the original biomolecules can be classified into four different categories:

- N- and C-terminally protected tripeptides with non-coordinating side chains /9/: Ac-ProLeuGly-BIMA and BIP-IleAlaGly-OEt (Scheme 2),
- N- and C-terminally protected tripeptides containing histidyl residues in all possible locations /11/: BOC-ProLeuHis-BIMA, BOC-ProHisGly-BIMA, BOC-HisLeuGly-BIMA, BIP-HisAlaGly-OEt, BIP-IleHisGly-OEt and BIP-IleAlaHis-OMe (Scheme 3),
- amino acid derivatives of BIMA containing free amino groups /10,14/: Gly-BIMA, Phe-BIMA and His-BIMA (Scheme 4),
- dipeptide derivatives of BIMA containing free amino terminus /15/: LeuGly-BIMA, GlyLeu-BIMA, PheGly-BIMA and AlaPro-BIMA (Scheme 5).

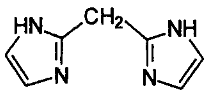

bis(imidazol-2-yl)methane
**BIM**

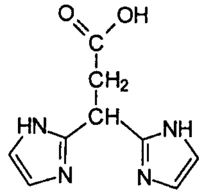

3-[bis(imidazol-2-yl)propionic acid
**BIP**

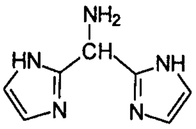

bis(imidazol-2-yl)methyl-amine
**BIMA**

Scheme 1

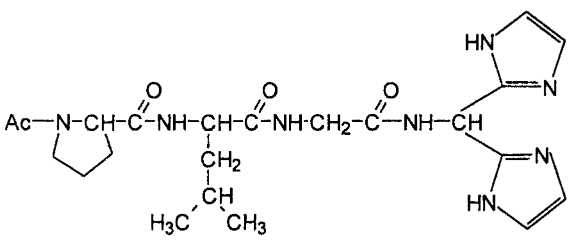

**Ac-ProLeuGly-BIMA**

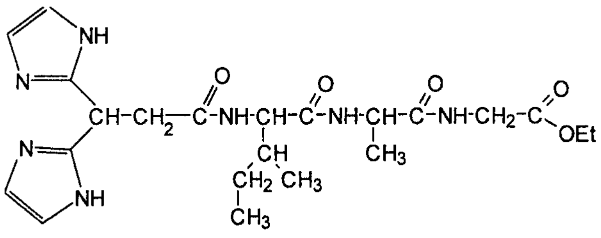

**BIP-IleAlaGly-OEt**

Scheme 2

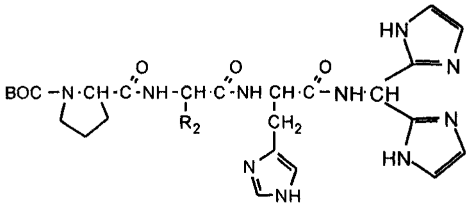

**BOC-ProLeuHis-BIMA**

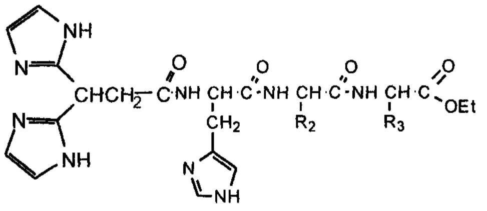

**BIP-HisAlaGly-OEt**

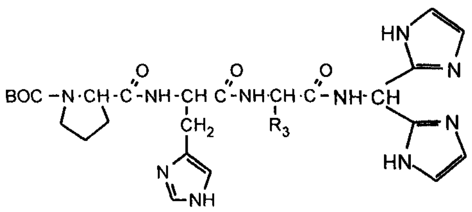

**BOC-ProHisGly-BIMA**

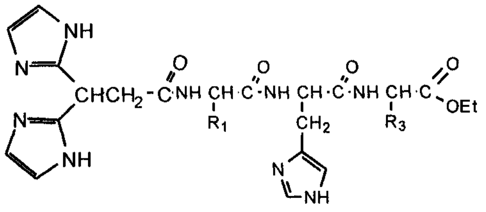

**BIP-IleHisGly-OEt**

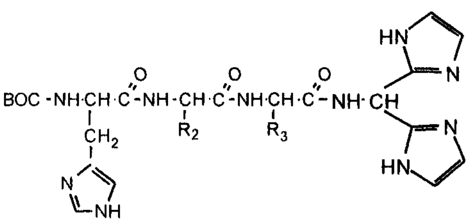

**BOC-HisLeuGly-BIMA**

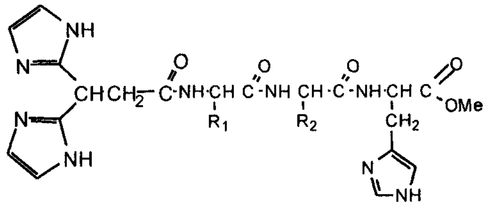

**BIP-IleAlaHis-OMe**

Scheme 3

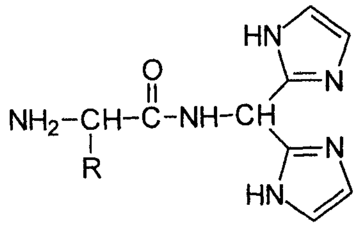

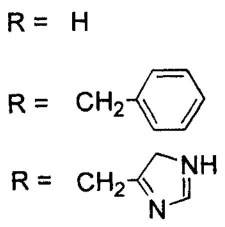

**Gly-BIMA**

**Phe-BIMA**

**His-BIMA**

Scheme 4

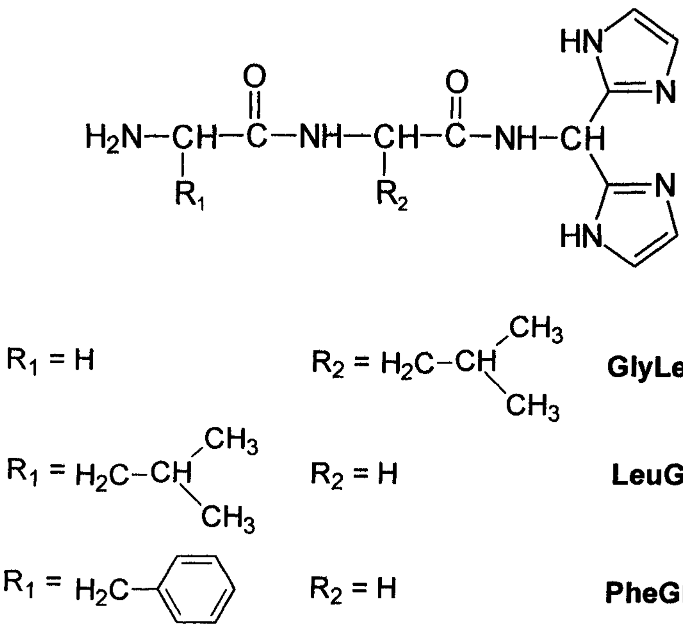

GlyLeu-BIMA

LeuGly-BIMA

PheGly-BIMA

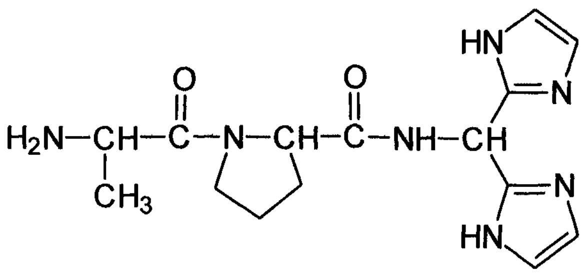

AlaPro-BIMA

Scheme 5

### Protonation equilibria of the ligands

The p*K* values of the bis(imidazol-2-yl) ligands were determined by potentiometric titrations and the values are summarized in Table 1.

**Table 1**

Protonation constants of the bis(imidazolyl) ligands. (T = 298 K, I = 0.2 mol/dm$^3$)

| Ligand | Im(1) | Im(2) | $NH_2$ | His(Im) | $COO^-$ | Ref. |
|---|---|---|---|---|---|---|
| BIM | 4.74 | 6.93 | | | | /9/ |
| BIP | 4.62 | 6.90 | | | 2.79 | /9/ |
| BIMA | – | 4.07 | 6.49 | | | /9/ |
| Ac-ProLeuGly-BIMA | 3.31 | 5.67 | | | | /9/ |
| BIP-IleAlaGly-OEt | 3.82 | 5.99 | | | | /9/ |
| BOC-ProLeuHis-BIMA | 2.85 | 5.25 | | 6.64 | | /11/ |
| BOC-ProHisGly-BIMA | 3.11 | 5.42 | | 6.38 | | /9/ |
| BOC-HisLeuGly-BIMA | 2.78 | 5.24 | | 6.65 | | /11/ |
| BIP-IleAlaHis-OMe | 3.73 | 5.84 | | 6.81 | | /11/ |
| BIP-IleHisGly-OEt | 4.01 | 5.67 | | 6.65 | | /9/ |
| BIP-HisAlaGly-OEt | 3.73 | 5.77 | | 6.77 | | /11/ |
| Gly-BIMA | 3.22 | 5.51 | 7.95 | | | /10/ |
| Phe-BIMA | 3.09 | 5.28 | 7.17 | | | /14/ |
| His-BIMA | 2.61 | 4.53 | 7.28 | 5.81 | | /14/ |
| GlyLeu-BIMA | 3.17 | 5.58 | 7.92 | | | /15/ |
| LeuGly-BIMA | 3.18 | 5.59 | 7.76 | | | /15/ |
| PheGly-BIMA | 3.19 | 5.61 | 7.33 | | | /15/ |
| AlaPro-BIMA | 2.97 | 5.52 | 8.11 | | | /15/ |

It is clear from Table 1 that the protonation of the nitrogen atoms of the bis(imidazolyl) residues always takes place in the acidic pH range and the presence of other protonation sites decreases the basicity (or p*K* values) of these nitrogen donors. As a consequence, the parent compound (BIM) has the highest p*K* values and the amino group in the close vicinity of the bis(imidazolyl) residue has the most significant influence on the basicity of the nitrogen donor atoms. In the case of BIMA only one p*K* value of the bis(imidazolyl) moiety lies in the measurable pH range ($pK > 1.5$), while in all other cases the differences in the basicities of the two nitrogen atoms are around 2 log units. It is also important to note that in the case of ligands containing terminal amino group and especially a third imidazole from histidyl residues the protonation equilibria of the 3 or 4 nitrogen atoms significantly overlap. The exact assignment of the protonation sites would require the determination of protonation microconstants. These values have not been determined yet, but the pH-dependent $^1H$ NMR studies on the ligand His-BIMA unambiguously revealed that the order of basicities of the 4 donor atoms follow the trend: N(Im) of BIM < N(Im) of His < N(amino).

### Formation of 4N-coordinated complexes

The results obtained for the copper(II) complexes reveal that the bis(imidazolyl) residues are the primary metal binding sites in the case of all ligands. The complex formation reactions generally start in strongly acidic solution (pH ≤ 2) and stable mono- and bis-(ligand) complexes are formed containing 6-membered chelate rings. The other donor functions (His(Im) and terminal amino groups) are non-coordinated and

**Table 2**

Stability constants of the copper(II) complexes of the bis(imidazolyl) coordinated 2N- and 4N-complexes.

| Ligand | log $K'_1$ | log $K'_2$ | log($K'_1/K'_2$) | log($K_1/K_2$) |
|---|---|---|---|---|
| BIM | 9.64 | 7.39 | 2.25 | 2.25 |
| Ac-ProLeuGly-BIMA | 8.65 | 6.59 | 2.06 | 2.06 |
| BIP-IleAlaGly-OEt | 8.92 | 6.60 | 2.32 | 2.32 |
| BOC-ProLeuHis-BIMA | 7.73 | 6.33 | 1.40 | 2.45 |
| BOC-ProHisGly-BIMA | 8.32 | 6.36 | 1.96 | 3.33 |
| BOC-HisLeuGly-BIMA | 8.02 | 6.13 | 1.89 | 6.17 |
| BIP-IleAlaHis-OMe | 8.03 | 6.13 | 1.90 | 5.22 |
| BIP-IleHisGly-OEt | 8.02 | 5.36 | 2.66 | 5.36 |
| BIP-HisAlaGly-OEt | 8.31 | 6.12 | 2.19 | 5.23 |
| Gly-BIMA | 9.16 | 6.58 | 2.58 | – |
| Phe-BIMA | 8.27 | 6.37 | 1.90 | – |
| His-BIMA | 6.20 | 4.82 | 1.38 | – |
| GlyLeu-BIMA | 7.84 | 6.45 | 1.39 | – |
| LeuGly-BIMA | 8.01 | 6.49 | 1.52 | – |
| PheGly-BIMA | 8.80 | 6.65 | 2.15 | – |
| AlaPro-BIMA | 9.34 | 6.61 | 2.73 | – |

protonated under these conditions and the stoichiometries of the various bis(imidazolyl)-coordinated species can vary between [CuL] and [$CuH_2L$], and [$CuL_2$] and [$CuH_4L_2$] for the mono- and bis-(ligand) complexes, respectively. The overall stability constants of these complexes (log $\beta_{pqr}$) have been reported in the previous publications /9-15/, but these values cannot be easily compared, because of the different protonation sites of the ligands. In Table 2 the log $K'_1$ and log $K'_2$ values are summarized, which represent the metal ion coordination of one or two bis(imidazolyl) residues and can be obtained from the overall stability constants by substracting the p$K$ values of the non-coordinated donor functions. The last column in Table 2 contains the ratio of the stability constants (log($K_1/K_2$)) of the species [CuL] and [$CuL_2$]. In the case of BIM, Ac-ProLeuGly-BIMA and BIP-IleAlaGly-OEt the two ratios, log($K'_1/K'_2$) and log($K_1/K_2$) are the same because extra protonation sites are not available, while the change of these values reflects the change of coordination geometries in all other cases.

It is clear from Table 2 that the ratios of the stability constants of the 2N- and 4N- coordinated complexes, log($K'_1/K'_2$), are around two log units in all cases. For the same coordination modes exact agreement of these values cannot be expected because there can be some differences in the p$K$ values of the side chain donor functions of the free and coordinated ligands. The existence of the same coordination mode of the bis(imidazolyl) residues is, however, clearly supported by the spectroscopic parameters of the various bis(ligand) complexes. The absorption maxima of the [$CuH_nL_2$] complexes are always in the range $\lambda$ =

590±15 nm, while the agreement of EPR parameters is even more pronounced – $g_{||}$ = 2.235±0.005 and $A_{||}$= 196±5 x $10^{-4}$ $cm^{-1}$. It is also important to emphasize that a well-resolved, 9-line $^{14}N$ superhyperfine splitting can be observed in the parallel region of EPR spectra /9/ for all species listed in Table 2 in agreement with the coordination of four equivalent nitrogen atoms as shown by Scheme 6.

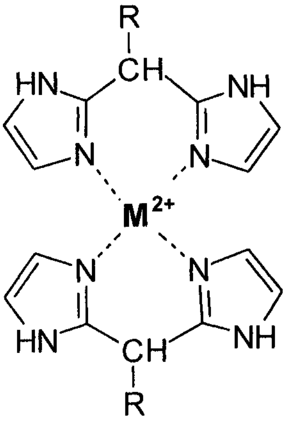

$[CuH_nL_2]^{(2+n)+}$

Scheme 6

The comparison of the $\log(K'_1/K'_2)$ and $\log(K_1/K_2)$ values in Table 2 reveals that the deprotonation of the non-coordinated donor functions of the ligands significantly influences the ratios of stepwise stability constants in most cases. These processes are generally accompanied by drastic changes of spectral parameters, suggesting the appearance of new coordination modes. In the case of amino acid and dipeptide derivatives these values cannot even be determined, because the deprotonation of the non-coordinated ammonium group is accompanied by the deprotonation and metal ion coordination of the amide functions and the species [CuL] and/or $[CuL_2]$ do not exist in measurable concentrations. For the terminally protected tripeptides containing His(imidazolyl) residues the deprotonation of the side chain donor functions can be easily followed and generally results in an increase in the ratio of stepwise stability constants. This can be explained by the tridentate coordination of the ligands in the species [CuL], which enhances the thermodynamic stability of the 1:1 complexes, but slightly suppresses bis(ligand) complex formation. It is also clear from Table 2 that the tridentate coordination is more preferred if the chelating agent is present at the N-termini (peptides of BIP). In the case of C-terminal derivatives (peptides of BIMA) the ratio of the stepwise stability constants increases as the distance between the chelating and monodentate side chains increases. As a consequence, the most stable 1:1 complex was obtained with BOC-HisLeuGly-BIMA and the outstanding stability of this species was explained by the equatorial coordination of the histidyl residue in the form of 16-membered macrochelate as shown by Scheme 7.

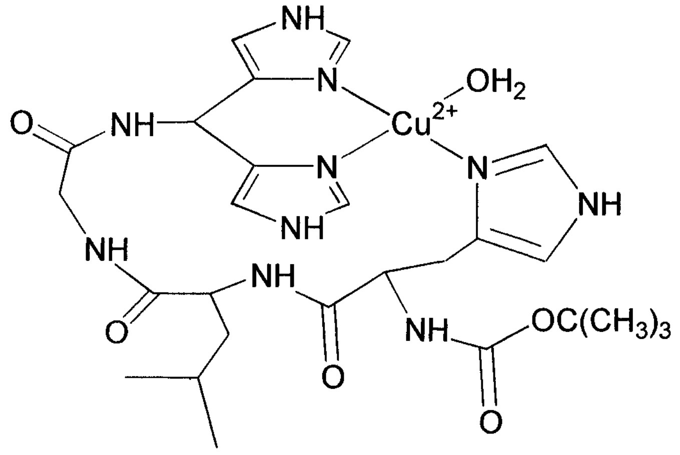

**$[CuL]^{2+}$ L = BOC-HisLeuGly-BIMA**

Scheme 7

The tridentate nature and the equatorial coordination of the ligand is supported by the change of the spectral parameters, too. The formation of [CuL] from [CuHL] is accompanied by a significant blue shift of absorption maxima (from 685 nm to 635 nm) in the copper(II)–BOC-HisLeuGly-BIMA system. The variation of EPR parameters reveals similar tendencies: $g_{||}$ = 2.27 and 2.30 and $A_{||}$= 176 and 172 x $10^{-4}$ $cm^{-1}$ for [CuL] and [CuHL], respectively.

Deprotonation of the bis(ligand) complexes, namely the formation of $[CuL_2]$ from $[CuH_nL_2]$ is accompanied by the opposite change of spectral parameters. For example, in the case of the copper(II)–BOC-HisLeuGly-BIMA system the deprotonation of the histidyl residues resulted in a 41 nm red shift of absorption spectra (from 595 nm to 636 nm) and a small decrease of EPR hyperfine coupling constants (from 197 x $10^{-4}$ $cm^{-1}$ to 191 x $10^{-4}$ $cm^{-1}$). These parameters are in agreement with the axial coordination of at least one of the side chain histidyl residues in the $[CuL_2]$ species. Similar spectral changes were observed for all other ligands and both the thermodynamic and spectral data suggest that the extent of axial coordination increases with the increase of the distance between the chelating bis(imidazolyl) and monodentate histidyl side chains. It is also important to note that extra deprotonation reactions were not observed in the copper(II) complexes of any N-protected tripeptides. As a consequence, the bis(imidazolyl) agents are the primary metal binding sites of these ligands at all pH values, but the side chain donor functions may enhance the metal binding ability of these ligands *via* equatorial or axial coordinations.

### Ligand and/or imidazole bridged dinuclear complexes

The presence of the terminal amino groups in the bis(imidazolyl) derivatives of amino acids and dipeptides makes the complex formation processes of these ligands much more complicated than those with the terminally protected ones. This is demonstrated by Figure 1 where the speciation curves obtained in the copper(II)–Gly-BIMA, His-BIMA and GlyLeu-BIMA systems are plotted in equimolar solutions.

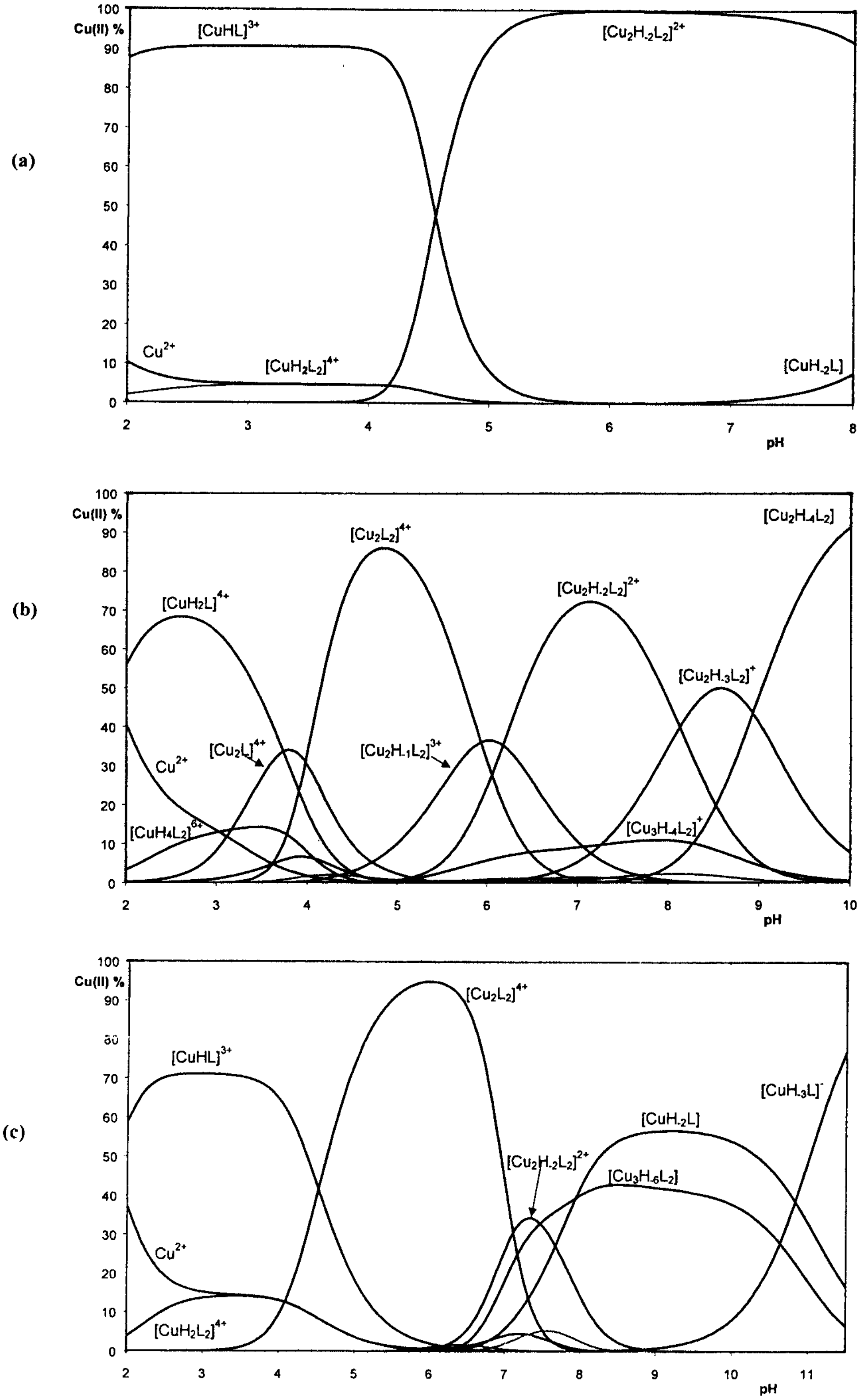

**Fig. 1:** Concentration distribution of the complexes formed in equimolar solution of copper(II) and Gly-BIMA (a), His-BIMA (b) and LeuGly-BIMA (c) as a function of pH. ($c_{Cu(II)} = c_L = 4 \times 10^{-3}$ mol/dm$^3$).

It is clear from Figure 1 that the deprotonation of the non-coordinated ammonium groups results in the formation of various dinuclear complexes in slightly acidic solutions in all cases. In the case of His-BIMA and the dipeptide derivatives, the stoichiometry of the first dimeric species is $[Cu_2L_2]$ suggesting that the amide nitrogen atoms are not yet metal binding sites in these species. The formation of $[Cu_2L_2]$ is accompanied by a blue shift of absorption maxima and the appearance of broad, unresolved EPR spectra. The species are not EPR silent, but the line broadening suggests a dipolar interaction between copper(II) ions and the presence of a mixture of copper(II) ions with different coordination environments. These spectral changes can be explained by the assumption of the structures shown by Schemes 8.a,b and 9.a,b for His-BIMA and dipeptide-BIMA systems, respectively.

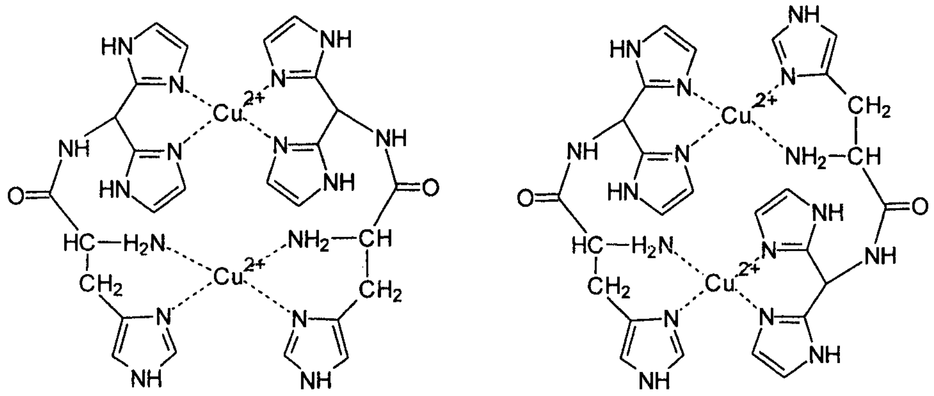

(a) (b)

$[Cu_2L_2]^{4+}$

Scheme 8

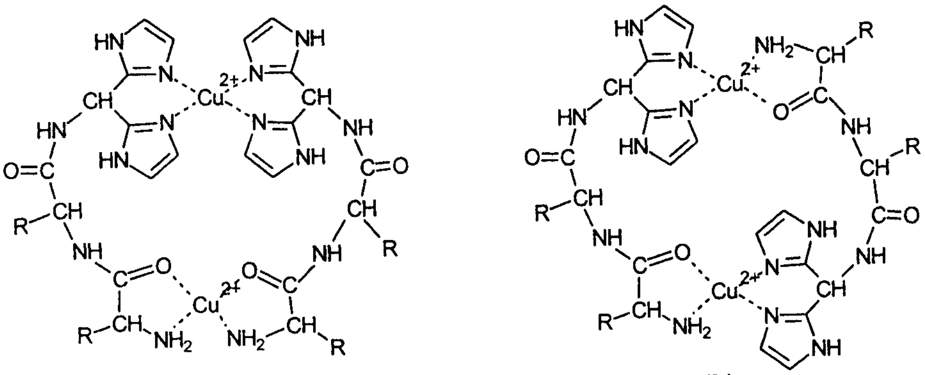

(a) (b)

$[Cu_2L_2]^{4+}$

Scheme 9

Both His-BIMA and dipeptide-BIMA ligands contain two, separated chelating sites in the molecules: the bis(imidazolyl) sites and the ($NH_2$,His(Im)) or ($NH_2$,CO) sites, respectively. Steric requirements rule out the coordination of these chelating sites to the same metal ion in a mononuclear complex, but the formation of ligand bridged dimeric complexes is possible in all cases. Two different isomeric forms of [$Cu_2L_2$] can be obtained by these types of coordinations. Schemes 8.a and 9.a correspond to the symmetrical arrangement of the donor sites, while 8.b and 9.b to the asymmetrical ones. Previous results on the factors influencing mixed ligand complex formation /21/ indicate the preference of symmetrical isomers and it is further supported by the results of the EPR measurements. The spectral parameters of the individual coordination modes cannot be determined, because of the high similarity of the overlapping species, but the observation of the $^{14}N$ superhyperfine splitting in the parallel region is a strong indication of the symmetrical coordination of the chelating sites.

An increase of pH results in the formation of another dinuclear complex with the stoichiometry of [$Cu_2H_{-2}L_2$]. This species was formed with all amino acids including Gly-BIMA and Phe-BIMA and dipeptide derivatives, except AlaPro-BIMA. Taking into account the pH range of deprotonation (pH $\leq$ 5) and the blue shift of the absorption maxima, the extra base consuming process cannot be hydroxo complex formation, but it should come from the deprotonation and metal ion coordination of the amide functions of the molecules. The spectral parameters obtained for the [$Cu_2H_{-2}L_2$] species of the bis(imidazolyl) derivatives of dipeptides and amino acids are significantly different, suggesting different metal binding modes. In the case of copper(II)–GlyLeu-BIMA system (or the other dipeptides) the shift of the absorption maxima from 627 nm to 546 nm upon the formation of [$Cu_2H_{-2}L_2$] from [$Cu_2L_2$] clearly indicates the increase of the number of coordinated nitrogen atoms. It can be best explained by the conversion of the ($NH_2$,CO) chelate to the ($NH_2$,$N^-$) chelate in the same ligand bridged structure as shown by Scheme 9.a,b. The species [$Cu_2H_{-2}L_2$] was not formed in the case of AlaPro-BIMA containing the secondary amide bond between Ala and Pro residues and this observation provides further support that the amide nitrogen next to the amino groups takes place in metal binding in these species.

In the case of the copper(II)–amino acid-BIMA systems (Gly-BIMA, Phe-BIMA and His-BIMA) the absorption maxima of the species [$Cu_2H_{-2}L_2$] occur at 590±5 nm and the EPR spectra are characteristic of dimeric copper(II) complexes with relatively short copper(II)–copper(II) distances. These parameters can be explained by the tridentate, [$NH_2$,$N^-$,N(Im)]-coordination of each ligand containing a bridging imidazole residue at the fourth equatorial coordination site (Scheme 10).

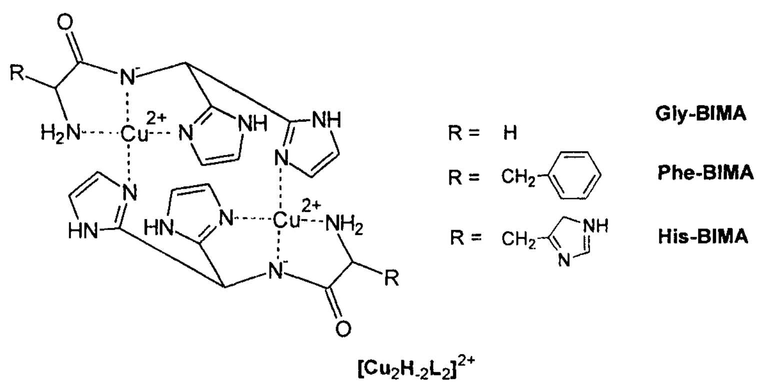

Scheme 10

The EPR parameters obtained from the perpendicular signals of the spectra made it possible to calculate the Cu–Cu distances in the dinuclear species and the values 390, 393 and 397 nm were obtained for Gly-BIMA, Phe-BIMA and His-BIMA, respectively.

### Complexes with negatively charged imidazolato residues

The increase of pH results in one or two more extra base consuming processes above pH 7 in all types of systems depicted in Figure 1. The stoichiometries of the various species and the binding sites of the ligands are, however, significantly different in the three cases. For copper(II)–Gly-BIMA (or Phe-BIMA) systems the deprotonation and formation of $[CuH_{-2}L]$ were explained by hydroxo complex formation, which is followed by precipitation of metal hydroxide at high pH values. These observations suggest that the bridging imidazole residues are not able to prevent hydrolytic reactions in alkaline solution. However, in the case of the copper(II)–His-BIMA system the EPR spectra provided an unambiguous proof for the existence of dimeric complexes at any pH values in the basic range. The stoichiometries of these dinuclear species can be given as $[Cu_2H_{-3}L_2]$ and $[Cu_2H_{-4}L_2]$ and their formation is accompanied by a slight blue shift of the absorption band (from 592 nm to 565 nm). The Cu–Cu distance can be calculated from the perpendicular signal of EPR spectra at pH 10.4 and a small decrease of this value (from 397 pm to 384 pm) was obtained. The changes of these parameters strongly support the deprotonation of the pyrrole type N(1)H group of the coordinated imidazole functions. The deprotonation results in a negatively charged imidazolato group and its metal binding is generally accompanied by a slight decrease of metal–ligand bonds and blue shift of the absorption maxima of copper(II) complexes. Metal ion induced deprotonation of the imidazole-N(1)H groups have already been reported for many peptide complexes containing histidyl residues /22-28/. Palladium(II) and gold(III) were reported to be especially effective in the promotion of ionisation /26-28/, while p*K* values around 10 were reported for copper(II) complexes /22-25/. The p*K* values obtained for the successive deprotonation of the complex $[Cu_2H_{-2}L_2]$ of His-BIMA are 8.13 and 8.93 /14/. These values are slightly lower than those reported for copper(II) complexes in the literature, but these species have different charges and the charge neutralization probably has a significant contribution to the outstanding thermodynamic stability of the species $[Cu_2H_{-4}L_2]$. It is also important to note that, in principle, similar deprotonation reactions could occur in the copper(II)–Gly-BIMA or Phe-BIMA systems, too. In the case of these ligands the extra deprotonation reactions were, however, interpreted by the hydrolysis of the metal ions. The differences between the reaction of His-BIMA and the other two amino acids probably can be explained by the weak axial interaction of the side chain histidyl imidazole donor groups. This axial interaction enhances the metal binding ability of His-BIMA and, at the same time, suppresses the chance of hydrolytic reactions.

The dipeptide derivatives of BIMA contain two amide functions which are in the position to form joined chelate rings with the terminal amino and bis(imidazolyl) nitrogen donor atoms. As a consequence, the dinuclear complex $[Cu_2H_{-2}L_2]$ is transformed to a mononuclear copper(II) species $[CuH_{-2}L]$ above pH 7 in equimolar solution. This complex predominates in the pH range 8 to 10 and its spectral parameters are in a very good agreement with those of the 4N-coordinated peptide complexes of copper(II) /29/. The absorption maxima of the $[CuH_{-2}L]$ complexes of GlyLeu-BIMA, LeuGly-BIMA and PheGly-BIMA were measured at $\lambda$ = 513-514 nm and the EPR parameters were obtained as follows: $g_{||}$ = 2.174 to 2.178 and $A_{||}$ = 204 to 205 x

$10^{-4}$ $cm^{-1}$. These parameters correspond well to the simultaneous coordination of 4N donor atoms as shown by Scheme 11.a.

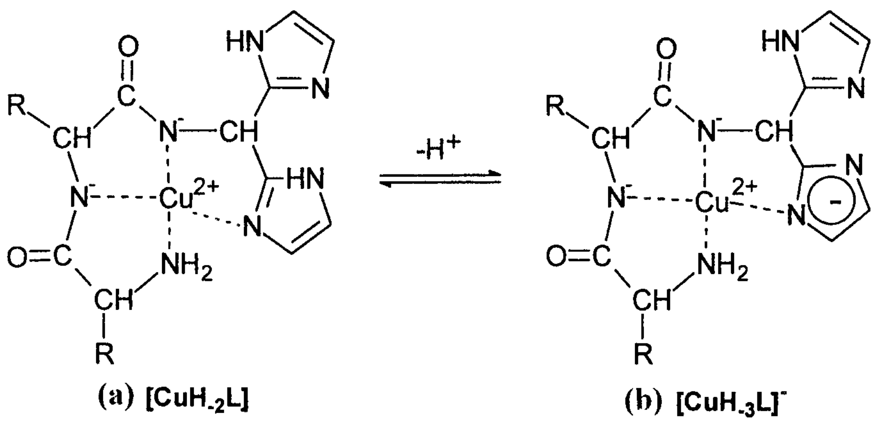

Scheme 11

Another base consuming process takes place above pH 10, which may correspond to the deprotonation of the N(1)H group of the coordinated imidazole. The p*K* values of this process are 10.69, 10.75 and 10.51 for GlyLeu-BIMA, LeuGly-BIMA and PheGly-BIMA, respectively. These values are higher than those reported for the same process of His-BIMA, but in the case of the dipeptides the deprotonation results in negatively charged species, while even the final species $[Cu_2H_{-4}L_2]$ is neutral for His-BIMA. The deprotonation reactions are accompanied by small changes of spectral parameters, e.g.: $\lambda$ = 508 nm, $g_{||}$ = 2.168 and $A_{||}$= 209 x $10^{-4}$ $cm^{-1}$ were obtained for the $[CuH_{-3}L]$ species of LeuGly-BIMA supporting that the 4N-coordination mode remains intact in the species $[CuH_{-3}L]$ (see Scheme 11.b).

The multidentate character of the dipeptide ligands made it possible to bind more than one metal ion and it is important to note that the N(1)H deprotonation creates a new chelating site, too. As a consequence, precipitation was not observed even in slightly basic solution up to 2:1 = Cu(II):L ratio and the formation of various polynuclear complexes was suggested. It is clear from Figure 1 that the species $[Cu_2H_{-3}L]$, $[Cu_3H_{-6}L_2]$ and $[Cu_4H_{-8}L_2]$ are the major polynuclear complexes in alkaline solutions and their formation is represented by Scheme 12. Following the binding of the second amide group in $[CuH_{-2}L]$ the imidazole nitrogen atoms get to a sterically favourable position which promotes the deprotonation of a pyrrole type N(1)H group resulting in the formation of $[Cu_2H_{-3}L]$ species (Scheme 12.a). It is also clear from the structure of $[Cu_2H_{-3}L]$ that the coordination sphere of one metal ion is not saturated yet, and this results in formation of tri- or tetranuclear complexes depending on the metal to ligand ratio. At 3:2 metal to ligand ratio the trinuclear complex $[Cu_3H_{-6}L_2]$ predominates above pH 7, in which all copper(II) ions are coordinated by 4 N donor atoms (Scheme 12.b), while at 2:1 metal to ligand ratio a mixed hydroxo complex $[Cu_4H_{-8}L_2]$ is formed connecting the $[Cu_2H_{-3}L]^+$ moieties *via* hydroxo bridges (Scheme 12.c).

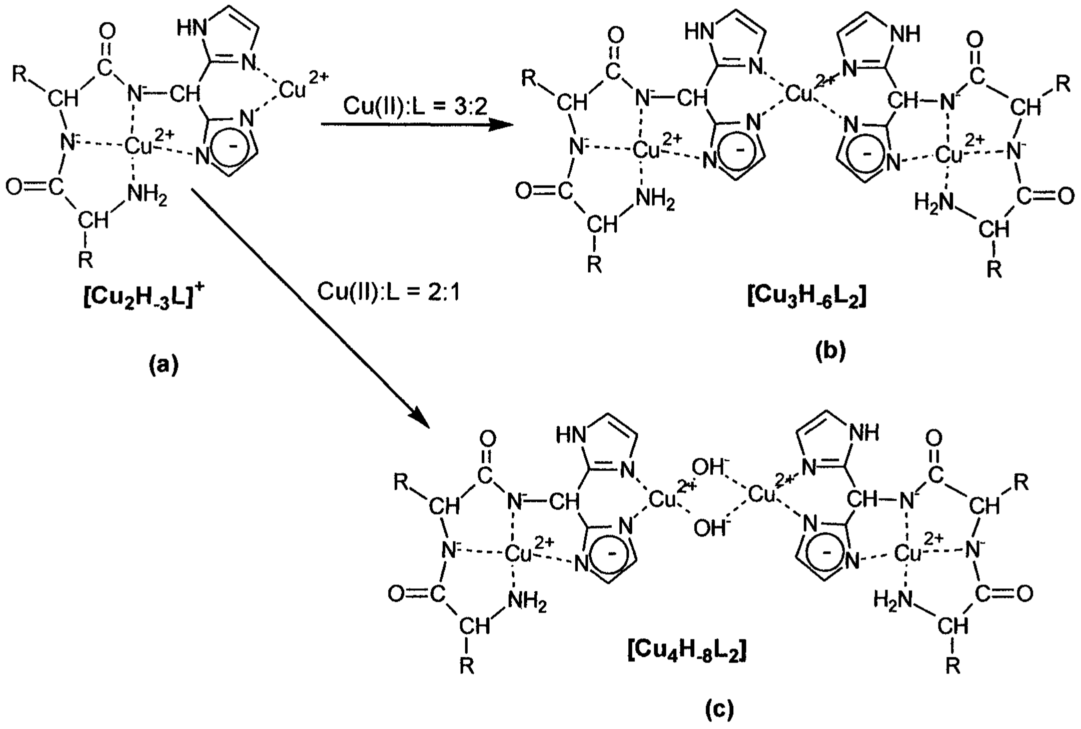

Scheme 12

The existence of the trinuclear complexes was supported by MALDI-MS spectroscopic measurements, too. The mass spectra were reported in a previous publication /15/ and they unambiguously prove the presence of three copper(II) ions in the complex formed at pH 8 in solution containing copper(II) and LeuGly-BIMA ligand at 3 to 2 ratio. The isotope distribution pattern obtained for the complex is in very good agreement with that calculated for the molecule ion $[Cu_3H_{-6}L_2]H^+$ = $[Cu_3C_{30}H_{40}O_4N_{14}]H^+$. There is a difference, however, between the estimated $(M([Cu_3C_{30}H_{40}O_4N_{14}]H^+)$ = 852.3) and measured (M = 855.3) molecular weights, which correspond to the uptake of three protons. The higher values for the measured molecular weight could be explained by the reduction of $Cu^{2+}$ to $Cu^+$ during the MS ionization and flight /30,31/.

### CONCLUDING REMARKS

The results obtained for the copper(II) complexes of amino acids and peptides reveal that bis(imidazolyl) analogues of these biomolecules are very effective ligands for metal binding. The nitrogen donor atoms of the chelating bis(imidazolyl) residues are the major metal binding sites under acidic conditions (Scheme 6) and they remain the exclusive metal binding sites if the terminal amino group or an effective side chain donor function are not present in the molecules. The high thermodynamic stability of the metal complexes of the bis(imidazolyl) ligands render these ligands as potential enzyme inhibitors. Moreover, specific enzyme inhibitors may be obtained by attaching the bis(imidazolyl) ligands to the preferred peptide sequence for the enzyme cleavage.

The terminal amino group can be considered as another anchor for metal binding with these ligands. The multidentate character of these ligands results in the formation of various polynuclear complexes including the ligand bridged dimeric species (Scheme 8 and 9) and the imidazole bridged dimeric species (Scheme 10). These complexes can be considered as interesting structural models of the various dinuclear species of transition elements.

Finally, the most intriguing feature of the coordination chemistry of these ligands is that the deprotonation of the coordinated imidazole-N(1)H groups results in the appearance of a new chelating site in the molecules. It leads to the formation of stable trinuclear complexes *via* negatively charged imidazolato bridges and these species can be considered as promising structural and/or functional models of various metalloenzymes.

### ACKNOWLEDGEMENTS

The authors wish to thank Dr Helga Süli-Vargha (Research Group of Peptide Chemistry, Hungarian Academy of Sciences, H-1518 Budapest, Hungary) for the synthesis of ligands, Prof. Giovanni Micera and Dr. Daniele Sanna (Department of Chemistry, University of Sassari, Italy) for the EPR measurements and Prof. Miklós Zsuga and Dr. Sándor Kéki (Department of Applied Chemistry, University of Debrecen, Hungary) for MS measurements. The work was supported by the Hungarian Scientific Research Fund (OTKA T29141, and TS040685).

### REFERENCES

1. H. Sigel and R.B. Martin, *Chem. Rev.* **82**, 385 (1982).
2. I. Sóvágó, Metal Complexes of Peptides and Derivatives, in: *Biocoordination Chemistry* (ed. K. Burger), Ellis Horwood, Chichester , 1990; pp. 135-184.
3. H. Kozlowski, W. Bal and T. Kowalik-Jankowska, *Coord. Chem. Rev.* **184**, 319 (1999).
4. P. Tsiveriotis and N. Hadjiliadis, *Coord. Chem. Rev.* **190-192**, 171 (1999).
5. P. Tsiveriotis, G. Malandrinos and N. Hadjiliadis, *Reviews Inorg. Chem.* **20**, 305 (2000).
6. G.J.A.A. Koolhaas, W.L. Driessen, J. Reedijk, J.L. van der Plas, R.A.G. de Graaff, D. Gatteschi, H. Kooijman and A.L. Spek, *Inorg. Chem.* **35**, 1509 (1996).
7. T. Gajda, R. Krämer and A. Jancsó, *Eur. J. Inorg. Chem.* 1635 (2000).
8. C.J. Campbell, W.L. Driessen, J. Reedijk, W. Smeets and L.A. Spek, *J. Chem. Soc., Dalton Trans.* 2703 (1998).
9. K. Várnagy, I. Sóvágó, K. Ágoston, Z. Likó, H. Süli-Vargha, D. Sanna and G. Micera, *J. Chem. Soc., Dalton Trans.* 2939 (1994).
10. K. Várnagy, I. Sóvágó, W. Goll, H. Süli-Vargha, G. Micera and D. Sanna, *Inorg. Chim. Acta* **283**, 233 (1998).

11. K. Várnagy, I. Sóvágó, H. Süli-Vargha, D. Sanna and G. Micera, *J. Inorg. Biochem.* **81**, 35 (2000).

12. K. Ősz, K. Várnagy, I. Sóvágó, L. Lennert, H. Süli-Vargha, D. Sanna and G. Micera, *New J. Chem.* **25**, 700 (2001).

13. I. Sóvágó, K. Várnagy and K. Ősz, *Comments Inorg. Chem.* **23**, 149 (2002).

14. K. Ősz, K. Várnagy, H. Süli-Vargha, D. Sanna G. Micera and I. Sóvágó, *Inorg. Chim. Acta* **339**, 373 (2002).

15. K. Ősz, K. Várnagy, H. Süli-Vargha, D. Sanna G. Micera and I. Sóvágó, *J. Chem. Soc., Dalton Trans.* 2009 (2003).

16. Z. Likó and H. Süli-Vargha, *Tetrahedron Letters* **34**, 1673 (1993).

17. C.N.C. Drey and J.S. Fruton, *Biochemistry* **4**, 1 (1965).

18. C.N.C. Drey and J.S. Fruton, *Biochemistry* **4**, 1258 (1965).

19. C.C. Tang, D. Davalian, P. Huang and R. Breslow, *J. Am. Chem. Soc.* **100**, 3918 (1978).

20. M.S. Mohan, *Ind. J. Chem., Sect. A.* **20**, 252 (1981).

21. I. Sóvágó and A. Gergely, *Inorg. Chim. Acta* **37**, 233 (1979).

22. P.J. Morris and R.B. Martin, *J. Inorg. Nucl. Chem.* **33**, 2913 (1971).

23. R.J. Sundberg and R.B. Martin, *Chem. Rev.* **74**, 471 (1974).

24. I. Sóvágó, T. Kiss and A. Gergely, *J. Chem. Soc., Dalton Trans.* 964 (1978).

25. I. Sóvágó, E. Farkas and A. Gergely, *J. Chem. Soc., Dalton Trans.* 2159 (1982).

26. M. Wienken, B. Lippert, E. Zangrando and L. Randaccio, *Inorg. Chem.* **31**, 1983 (1991).

27. M. Wienken, E. Zangrando, L. Randaccio, S. Menzer and B. Lippert, *J. Chem. Soc., Dalton Trans.* 3349 (1993).

28. S.L. Best, T.K. Chattopadhyay, M.I. Djuran, R.A. Palmer, P.J. Sadler, I. Sóvágó and K. Várnagy, *J. Chem. Soc., Dalton Trans.* 2587 (1997).

29. I. Sóvágó, D. Sanna, A. Dessi, K. Várnagy and G. Micera, *J. Inorg. Biochem.* **63**, 99 (1996).

30. C.K.L. Wong and T.-W.Dominic Chan, *Rapid Commun. Mass Spectr.* **11**, 513 (1997).

31. P. Lubal, M. Kyvala, P. Hermann, J. Holubova, J. Rohovec, J. Havel and I. Lukes, *Polyhedron* **20** 47 (2001)

